# Seasonal variability of groundwater level effects on the growth of *Carex cinerascens* in lake wetlands

**DOI:** 10.1002/ece3.5926

**Published:** 2019-12-17

**Authors:** Wenjuan Feng, Pierre Mariotte, Ligang Xu, Alexandre Buttler, Luca Bragazza, Jiahu Jiang, Mathieu Santonja

**Affiliations:** ^1^ Key Laboratory of Watershed Geographic Sciences Nanjing Institute of Geography and Limnology Chinese Academy of Sciences Nanjing China; ^2^ University of Chinese Academy of Sciences Beijing China; ^3^ Laboratory of Ecological Systems (ECOS) School of Architecture, Civil and Environmental Engineering (ENAC) Ecole Polytechnique Fédérale de Lausanne (EPFL) Lausanne Switzerland; ^4^ Swiss Federal Institute for Forest, Snow and Landscape Research (WSL) Lausanne Switzerland; ^5^ Grazing Systems Group Agroscope Nyon Switzerland; ^6^ Laboratoire de Chrono‐Environnement UMR CNRS 6249 UFR des Sciences et Techniques Université de Franche‐Comté Besançon France; ^7^ Field‐Crop Systems and Plant Nutrition Research Division Plant Production Systems Agroscope Nyon Switzerland; ^8^ Aix Marseille Univ Avignon Université CNRS IRD IMBE Marseille France

**Keywords:** groundwater, hydrological condition, plant growth, plant reproduction, plant species composition, Poyang Lake, wetlands

## Abstract

Groundwater level is crucial for wetland plant growth and reproduction, but the extent of its effect on plant growth can vary along with changed precipitation and temperature at different seasons. In this context, we investigated the effect of two groundwater levels (10 cm vs. 20 cm depth) on growth and reproductive parameters of *Carex cinerascens*, a dominant plant species in the Poyang Lake wetland, during three seasons (spring, summer, and autumn) and during two consecutive years (2015 and 2016). *Carex cinerascens* showed low stem number, height, and individual and population biomass in summer compared to spring and autumn. 10 cm groundwater level was overall more suitable for plant growth resulting in higher stem height and biomass. However, the interactive effect between groundwater level and season clearly demonstrated that the effect of groundwater level on plant growth occurred mainly in autumn. After the withering of the plant population in summer, we observed that *C. cinerascens* growth recovered in autumn to similar values observed in spring only with 10 cm groundwater level. Consequently, we could deduce that lowering groundwater level in the studied Poyang Lake wetland will negatively impact *C. cinerascens* regeneration and growth particularly during the second growth cycle occurring in autumn. Additionally, our results showed that, independently of the season and groundwater level, population biomass of *C. cinerascens* was lower during drier year. Altogether, our findings suggest that water limitation due to both reduction in precipitation and decreased groundwater level during the year can strongly impact plant communities.

## Graphical abstract

We investigated the effect of groundwater level on growth and reproductive parameters of *Carex cinerascens*, a dominant plant species in the Poyang Lake wetland, during two consecutive years. We clearly demonstrated that the effects of groundwater level on plant growth occur mainly in autumn.

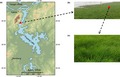

## 1 INTRODUCTION

Wetlands are important ecosystems that provide a diverse range of habitats for plants and animals (Dawson, Berry, & Kampa, 2013; Neckles, Murkin, & Cooper, 2010), and ensure several key ecosystem functions such as water purification, water storage, and shoreline stabilization (Costanza et al., 1997; Dawson et al., 2013). Several previous studies reported that hydrological processes control spatial and temporal heterogeneity of wetland vegetation and associated ecosystem functions (Garssen, Baattruppedersen, Voesenek, Verhoeven, & Soons, 2015; Renofalt, Merritt, & Nilsson, 2007; Valk, 2005; Webb, Wallis, & Stewardson, 2012). Water level affects seed germination, plant survival, growth, and reproductive parameters both directly (Keddy & Constabel, 1986; Kellogg, Bridgham, & Leicht, 2003; Raulings, Morris, Roache, & Boon, 2010) and indirectly through changes in soil nutrient availability or plant‐plant interactions (Sher & Marshall, 2003; Wilson & Keddy, 1986; Wolfe, Weishampel, & Klironomos, 2006).

Groundwater level is crucial for wetland development and maintenance, as it affects community assembly and composition (Deane et al., 2018; Hose, Bailey, Stumpp, & Fryirs, 2014; Valdez, Hartig, Fennel, & Poschlod, 2019) as well as the stability of plant communities (Ridolfi, D'Odorico, & Laio, 2006; Runhaar, Witte, & Verburg, 1997). Groundwater level is also a key factor determining plant growth and reproductive parameters (Booth & Loheide, 2012; Steed, Dewald, & Kolb, 2002) as the depth of the groundwater table strongly controls water, oxygen, and nutrient availability for plants (Kotowski, Andel, Diggelen, & Hogendorf, 2001; Muneepeerakul, Miralles‐Wilhelm, Tamea, Rinaldo, & Rodriguez‐Iturbe, 2008; Paul et al., 2003; Xu, Zhang, Tan, Li, & Wang, 2015). For example, Van der Valk and Baalman (2018) found that increasing groundwater level induced greater species richness and shoot density of meadow plant species in a mesocosm experiment. Li et al. (2014) reported higher plant biomass with increasing groundwater level in Dongting Lake wetland (China), while Scanga (2011) reported an opposite trend in Massachusetts (USA). In addition, Kotowski et al. (2001) showed that *Carex curta* and *Hydrocotyle vulgaris* biomasses did not respond to decreasing groundwater level in Netherlands.

Seasonal and annual climatic variations, along with hydrological fluctuations, can induce distinct plant responses to groundwater level (Dai et al., 2019; Runhaar et al., 1997) with cascading effect on vegetation distribution (Yuan, Wang, et al., 2017a). Thus, plant survival and growth under the same water level condition are not necessarily consistent at different seasons (Steed & DeWald, 2003). Surprisingly, previous studies testing the effects of groundwater level on plant growth were mostly conducted during few months and during one growing season only (*e.g.,* Hanke, Ludewig, & Jensen, 2015; Kotowski et al., 2001; Li et al., 2014). Climatic parameters like temperature and precipitation can strongly vary across the seasons (Xu et al., 2015) and some previous studies reported that the main hydrologic factors for plant growth and vegetation distribution differ between spring and autumn (Dai et al., 2019; Runhaar et al., 1997). However, to our knowledge, the extent to which variations in groundwater level could affect plant parameters during distinct plant growing periods (*i.e.,* spring vs. autumn) in relation to varying meteorological conditions remains poorly studied.

To fill this knowledge gap, we investigated the effect of two groundwater levels (10 cm vs. 20 cm depth) over three seasons (spring, summer, and autumn) on the growth and reproductive parameters of *Carex cinerascens* Kük., a dominant plant species in the Poyang Lake wetland (China), during two consecutive years (2015 and 2016). We hypothesized that: (a) *C. cinerascens* growth parameters are greater in spring and autumn (*i.e*., during the growing seasons) compared to summer; (b) 10 cm groundwater level favors both *C. cinerascens* growth and reproductive parameters as compared to 20 cm groundwater level; (c) the effect of groundwater level is also stronger in autumn than in spring and summer because lower autumn precipitation interacts with groundwater level to limit plant growth in the Poyang Lake wetland.

## 2 MATERIALS AND METHODS

### 2.1 Study site

**Figure 1:**
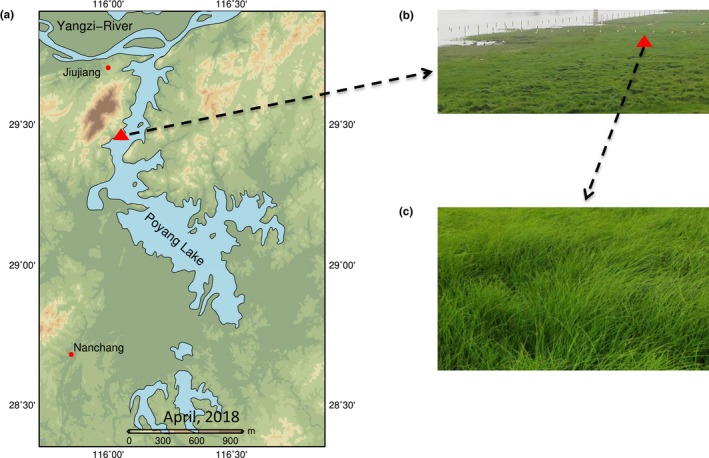
Map of the Poyang Lake (a) with the location of the Research Station (red triangle) and the sampled *Carex cinerascens* community (b, c). Photography credit: Junxiang Cheng

Poyang Lake (28.37 to 29.75°N, 115.78 to 116.75°E) is included in Yangtze River drainage basin and is currently the largest freshwater lake in China (Figure 1a). The climate is subtropical monsoon with a mean annual temperature of 17.6°C and mean annual precipitation of 1,528 mm. Precipitation increases from January reaching the maximum in June and decreases sharply from July to September with dry conditions lasting until December (Guo, Hu, & Jiang, 2008). Groundwater level of the lake is strongly controlled by the precipitation in the basin but also impacted by Three Gorges Dam and sand mining activities that regularly induce a decrease in water level (Lai et al., 2014; Zhang et al., 2012). In the Poyang Lake wetland, there are five main plant communities dominated by *Phragmites australis* (Cav.) Trin. ex Steud*., Triarrhena lutarioriparia* L. Liu*, Artemisia selengensis* Turcz. ex Bess.*, Carex cinerascens* Kük, and *Phalaris arundinacea* L. in a typical zonation from upland to the shoreline (Wang, Han, Xu, Wan, & Chen, 2014; You et al., 2015).

### 2.2 Plant material sampling

The experiment was conducted with *Carex cinerascens* Kük. (Figure 1b,c), a perennial rhizomatous species, which is a dominant and widely distributed hygrophyte plant species in the wetland zone, mostly subjected to groundwater level fluctuation in the Poyang lake wetland (Yuan, Yang, Liu, & Wang, 2017b; Zhang et al., 2019). *C. cinerascens* has a phenology characterized by two growing periods in spring and autumn, respectively. In spring, plants emerge with increasing temperature in February and they flower and fruit in April with a peak of biomass production at the end of April. Then, aboveground part of plants dies during the summer flooding. In autumn, after flooding recession, new stems are produced with a peak of biomass around October. Then the plants generally wither with decreasing temperature in winter and new stems sprout again in the following spring.

In February 2014, six turfs (38 cm diameter × 38 cm depth) were collected in a wetland area near the Poyang Lake Wetland Research Station (29.45°N, 116.06°E). The turfs contained both intact *C. cinerascens* individuals and corresponding undisturbed soil. Each turf was 30 cm depth because the roots of *C. cinerascens* never exceeded 30 cm depth, as observed during regular field investigations. Soil collected in this area had a pH of 6.3, a bulk density of 1.21 g/cm^3^, a total organic carbon (TOC) concentration of 27.19 g/kg, a total nitrogen (TN) concentration of 1.43 g/kg, and a total phosphorus (TP) concentration of 0.86 g/kg. At the same time, the subsoil below the sampled turfs was also collected and mixed to fill the devices used in the experiment (see below for explanation).

### 2.3 Experimental design

The experiment was established with two groundwater levels corresponding to a depth of 10 and 20 cm (3 replicates per treatment) and representing the distance from the soil surface to the free groundwater table. Groundwater levels were selected as representative of realistic water fluctuations in Poyang Lake wetland and linked to the rooting depth of *C. cinerascens*. Previous measurements of *C. cinerascens* root system showed that 85% of the root biomass is found in the first 10 cm (unpublished results), thus 10 cm groundwater level would slightly limit water access whereas 20 cm groundwater would correspond to a high water access limitation. We used acrylic glass containers (50 cm height × 38 cm diameter) that included three layers: 10 cm of coarse sand at the bottom to ensure the connectivity between the container and a water tank, 10 cm of mixed subsoil collected from the field covered by the 30 cm deep turf of *C. cinerascens* on the top. Containers were left outside at the Poyang Lake Wetland Research Station from February 2014 to December 2016, so that meteorological conditions were the same as the natural wetland (Appendix). Before the beginning of the groundwater level manipulation, turfs were watered with 2 L of tap water characterized by 0.16 mg L^−1^ of N‐NH_4_^+^, 0.65 mg L^−1^ of N‐NO_3_^−^, and 0.10 mg L^−1^ of P‐PO_4_^3+^. For the subsequent 7 days, we supplied the plants with 0.5 L of tap water twice daily (morning and afternoon) to acclimatize the plants.

After the week of plant acclimatization, the groundwater level was adjusted daily to the targeted depth (10 cm or 20 cm) with tap water. A valve was installed 5 cm from the bottom of each container. Through this valve, each container was connected to its water tank (25 cm height × 25 cm diameter) by a plastic pipe to adjust the groundwater level. Rulers were adhered to the surface of the containers to measure soil depth and water level in each container. The outside surface of each container was covered with aluminum foil to mimic underground darkness and prevent the temperature in the containers from rising excessively, and at the same time, to allow an easy checking of the inner groundwater level. When it was not raining, the valve was kept on and the groundwater level was adjusted manually every day to reach the desired groundwater levels. The valves were switched off before raining to reduce nutrient leakage, and the redundant water was drained after raining event.

### 2.4 Meteorological data

The temperature and precipitation data were obtained from the Poyang Lake Wetland Research Station. Since the plant growing duration in spring and autumn is around 3 months long, the average temperature and total precipitation of 3 months before plant traits measurement was used to indicate the meteorological conditions during plant growth (Table 1) corresponding to February 1st to April 30th for spring, May 1st to July 31st for summer, and August 1st to October 31st for autumn.

**Table 1:** Total precipitation and average temperature during the three months preceding plant measurement in spring, summer, and autumn

	Year	Spring	Summer	Autumn
Precipitation (mm)	2015	363	659	228
	2016	301	955	152
Temperature (°C)	2015	12.4	25.0	24.1
	2016	13.1	25.2	24.8

### 2.5 Plant growth parameters

Plant growth parameters of *C. cinerascens* were measured in 2015 and 2016 at the spring peak of biomass production (April), during summer before the autumn sprouting (July), and at the autumn peak of biomass production (October).

The stem number per container and the height (cm) of 5 random stems per container were measured. Three stems among the 5 randomly selected were cut at the soil surface, and oven‐dried weight (65°C for 72 hr) of the aboveground biomass (stem and leaves) was measured and used to determine mean aboveground biomass per individual. Aboveground biomass of the entire population of *C. cinerascens* per pot was estimated based on the mean individual biomass × stem number per pot. In addition to growth parameters, the number of flowering stems per pot was measured in April 2015 and 2016.

Other plant species, in addition to the dominant *C. cinerascens*, grew in containers over the 3‐year long experiment. Stem number of each auxiliary species was recorded in each container, and Shannon diversity index (H) was calculated as: $H=-\sum_{i=1}^{S}P_{i}\;\ln\;P_{i}$, where *P_i_* is fraction of the entire population made up of species *i* and *S* is the species number. In our experiment, the auxiliary species included *Artemisia selengensis* Turcz. ex Bess., *Conyza canadensis* L., *Mazus japonicus* (Thunb.) Kuntzw, *Ixeris chinensis* (Thunb.) Nakai, *Polypogon fugax* Nees ex Steud., *Echinochloa crusgalli* (L.) Beauv, *Ranunculus polii* Franch., *Alopecurus aequalis* Sobol., *Soliva anthemifolia* (Juss.) R. Br., *Daucus carota* L., and *Astragalus sinicus* L.

### 2.6 Data analysis

Statistical analyses were performed with the R software (version 3.3.1). Significance was evaluated in all cases at *p* < .05. Data were log‐ or square root‐transformed when necessary to meet the assumption of normality and homoscedasticity.

We used linear mixed‐effect models (*nlme* package), followed by Tukey HSD tests for post hoc comparisons, to examine the responses of *C. cinerascens* parameters and vegetation diversity to groundwater level (10 and 20 cm), season (spring, summer, and autumn), year (2015 and 2016) and their interactions. We treated groundwater level, season, year and their interactions as fixed factors, and container as random factor. The random part of the model allowed us to account for repeated measurements though time. Since the number of inflorescences of *C. cinerascens* was only recorded in spring, the effect of season was not tested on this parameter (*i.e.,* only groundwater level, year and their interactions).

## 3 RESULTS

### 3.1 Meteorological conditions

The mean temperature in 2016 was slightly higher than in 2015 in spring, summer, and autumn (Table 1). The precipitation in spring and autumn 2016 was lower than in 2015, while the precipitation in summer was higher in 2016 compared to 2015 (Table 1).

### 3.2 Response of growth parameters

**Figure 2:**
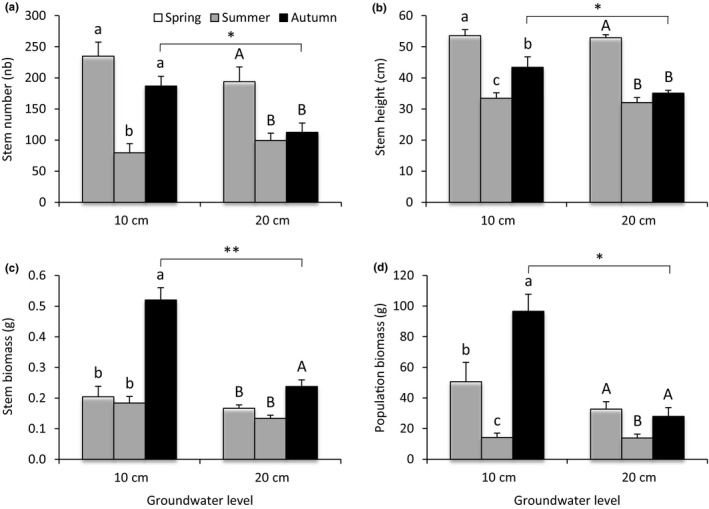
Stem number (a), stem height (b), stem biomass (c), and population biomass (d) of *C. cinerascens* in relation to groundwater level and season (Table 2). Values are mean ± *SE* (*n* = 6). Different letters indicate significant differences between seasons for the same groundwater level. Stars indicate significant difference between groundwater levels according to the season considered (* for *p* < .05, and ** for *p* < .01)

Stem number was 24% lower in 2016 compared to 2015 (Tables 2 and 3). The differences in stem number across seasons were dependent on the groundwater level considered (significant groundwater level × season interaction, Table 2). Highest number of stems was recorded in spring, then stem number decreased in summer and increased in autumn at 10 cm groundwater level, while the number of stems decreased in summer and remained constant in autumn at 20 cm groundwater level (Figure 2a). By consequence, we observed no difference in stem number between spring and autumn (i.e., the two growing seasons) at 10 cm groundwater level, but 73% less stems were observed in autumn compared to spring at 20 cm groundwater level (Figure 2a). The effect of groundwater level was also depended on the season considered, as we observed 66% more stems at 10 cm compared to 20 cm groundwater level only in autumn (Figure 2a).

**Table 2:** Effects of groundwater level (WL, 10 or 20 cm), season (spring, summer, and autumn), year (2015 and 2016), and their interactions on *C. cinerascens* growth parameters and plant community diversity. Inflorescence number was only recorded in spring so only WL, year and their interaction were tested for this parameter. *F*‐values and associated *p*‐values (with the respective symbols * for *p* < .05, ** for *p* < .01, and *** for *p* < .001) are indicated

	WL		Season		Year		WL × S		WL × Y		S × Y		WL × S × Y	
	*F*	*P*	*F*	*P*	*F*	*P*	*F*	*P*	*F*	*P*	*F*	*P*	*F*	*P*
Stem number	4.3		42.2	***	13.8	**	6.2	**	3.5		1.6		1.6	
Stem height	8.2	*	103.4	***	1.2		4.3	*	6.1	*	11.6	***	1.6	
Individual biomass	32.4	**	44.0	***	0.01		10.3	**	0.6		1.6		3.1	
Population biomass	9.8	*	36.7	***	4.3	*	8.4	**	0.1		1.9		2.2	
Plant diversity	0.4		6.8	**	19.2	***	2.1		1.7		6.2	**	2.7	
Inflorescence number	2.8				3.3		1.1							

**Table 3:** *Carex cinerascens* parameters and Shannon diversity index in relation to groundwater level, season, and sampling year. Values are means ± SE; *n* = 9 for groundwater level and year, *n* = 6 for season. Different letters indicate significant differences between treatments (post hoc Tukey tests)

	Groundwater level		Season			Year	
	10 cm	20 cm	Spring	Summer	Autumn	2015	2016
Stem number (nb)	167.1 ± 18.5 a	135.3 ± 13.9 a	214.4 ± 16.8 c	89.6 ± 9.3 a	149.6 ± 15.3 b	171.8 ± 18.8 b	130.6 ± 12.7 a
Stem height (cm)	43.5 ± 2.4 b	40 ± 2.3 a	53.2 ± 1 c	32.8 ± 1.2 a	39.2 ± 2.1 b	40.8 ± 2.1 a	42.7 ± 2.7 a
Individual biomass (g)	0.3 ± 0.04 b	0.18 ± 0.01 a	0.19 ± 0.02 a	0.16 ± 0.01 a	0.38 ± 0.05 b	0.24 ± 0.03 a	0.24 ± 0.04 a
Population biomass (g)	53.8 ± 9.8 b	24.9 ± 3.2 a	41.7 ± 7 b	14 ± 1.8 a	62.2 ± 11.9 b	45.6 ± 9.2 b	33.1 ± 6.4 a
Plant diversity (Shannon index)	0.57 ± 0.06 a	0.63 ± 0.07 a	0.57 ± 0.07 ab	0.74 ± 0.08 b	0.48 ± 0.07 a	0.47 ± 0.06 a	0.72 ± 0.06 b
Inflorescence number (nb)	35.7 ± 5.7 a	18 ± 6.6 a				20.8 ± 4.8 a	32.8 ± 8.4 a

**Figure 3:**
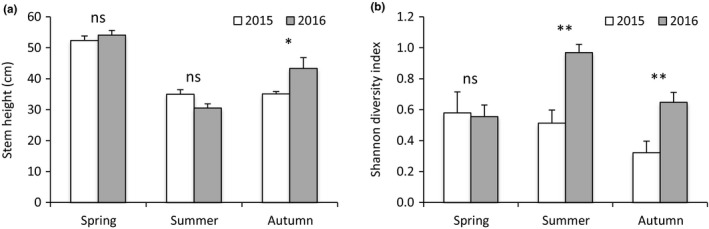
Stem height of *C. cinerascens* (a) and Shannon diversity index of the plant community (b) according to the season × year interaction (Table 2). Values are mean ± *SE*; *n* = 6. Stars indicate difference between years according to the season considered (* for *p* < .05, ** for *p* < .01, and ns for *p* > .05)

The stem height was 24% higher in 2016 compared to 2015 only in autumn (significant season × year interaction, Table 2; Figure 3a). As observed for stem number, groundwater level and season had an interactive effect on stem height (significant groundwater level × season interaction, Table 2). At 10 cm groundwater level, stem height decreased in summer and increased in autumn, but it reached a lower value compared to spring, while it decreased in summer and remained constant in autumn at 20 cm groundwater level (Figure 2b). By consequence, the difference in stem height between spring and autumn was more marked at 20 cm groundwater level (34%) compared to 10 cm groundwater level (19%). We also observed that in autumn plant height was 23.6% higher at 10 cm compared to 20 cm groundwater level (Figure 2b).

Individual aboveground biomass was higher in autumn compared to the two other seasons (Tables 2 and 3), but this difference was greater at 10 cm groundwater level (64%) compared to 20 cm groundwater level (38%), leading to difference between groundwater levels only in autumn (significant groundwater level × season interaction, Table 2; Figure 2c).

The aboveground biomass of *C. cinerascens* population was 27% lower in 2016 compared to 2015 (Tables 2 and 3). The aboveground biomass of *C. cinerascens* population at 10 cm groundwater level was two times higher compared to 20 cm groundwater level, and this difference was due to lower biomass in autumn at 20 cm compared to 10 cm groundwater level (significant groundwater level × season interaction, Table 2; Figure 2d). At 10 cm groundwater level, population biomass decreased from spring to summer and increased in autumn, reaching a value twice higher than in spring, while at 20 cm groundwater level it decreased in summer and increased in autumn to reach similar value as in spring (Figure 2d).

No significant effects of groundwater level, sampling year, or their interaction were observed in spring for inflorescence number (Table 2), despite a decreasing trend with decreasing groundwater level (Table 3).

### 3.3 Plant species diversity response

In contrast to growth parameters, plant species diversity was not affected by groundwater level (Tables 2 and 3). Plant species diversity was higher in 2016 compared to 2015, but this increase was dependent on the season (significant season × year interaction, Table 2), with higher values in summer and autumn (Figure 3b). The interaction between season and year also showed that there was no difference in plant species diversity across seasons in 2015, while the value in summer was higher than those in spring and autumn in 2016 (Figure 3b).

## 4 DISCUSSION

Our study aimed at testing the effects of groundwater level and interannual climatic variability on the growth parameters of *C. cinerascens*. In accordance with our first hypothesis, all measured *C. cinerascens* growth parameters showed lowest values in summer compared to spring and autumn. After reaching a peak of biomass in April, *C. cinerascens* started to wither until new plants emerged in August, corresponding to the second growing period (*see* Section 2.2. for further details). The summer quiescence of the species suggests that precipitation and temperature conditions in summer were not favorable for *C. cinerascens* growth (Table 1 and Appendix). *C. cinerascens* had higher stem number and height in spring compared to autumn, but similar population biomass during these two growing periods. Such results were consistent with those of Wang, Xu, Wan, and Chen (2016), but contradictory to those of Hu, Wu, Yao, and Xu (2015), which showed higher biomass of *Carex*‐dominated community in spring in Poyang Lake wetland. Both our study site and the study site of Wang et al. (2016) are located in the same area (northwest side of the lake) and have similar meteorological conditions*.* By contrast, the study site of Hu et al. (2015) is located at the south of the Lake, and during their experiment there was much more precipitation in spring compared to autumn, which can explain the higher biomass production in spring at this site. During our experiment, *C. cinerascens* also exhibited lower stem number and population biomass in 2016, which coincided with lower precipitations for both growing periods (spring and autumn) in comparison to 2015 (Table 1). Altogether, such findings further highlight the strong impact of precipitation in driving *C. cinerascens* productivity in Poyang Lake wetland.

Even though *C. cinerascens* withered in summer with fewer stem number compared to spring and autumn, some auxiliary species like *Artemisia selengensis* still grew and other auxiliary species like *Conyza canadensis* thrived due to the luxurious seed bank available in the Poyang Lake wetland (Wall & Stevens, 2015; Yu & Yu, 2011). In 2015, there was no difference in plant species diversity among the three seasons whereas in 2016, the wetter and warmer summer benefited the growth of auxiliary species. By consequence, plant species diversity increased in summer 2016 and it was higher than in spring and autumn 2016, as well as summer 2015. In 2016, the high plant species diversity remained until autumn, because the main auxiliary species *A. selengensis* and *C. canadensis* continued to grow until withering in winter, in spite of the recovery of *C. cinerascens*.

In accordance with our second hypothesis, 10 cm groundwater level was more beneficial for *C. cinerascens* growth compared to 20 cm groundwater level. Indeed, *C. cinerascens* exhibited higher stem height, individual, and population biomasses with groundwater closer to soil surface. This result is consistent with the findings of Li et al. (2014), which showed that *Carex brevicuspis* grows better in 20 cm than 40 cm groundwater level in Doting Lake wetlands, China. While increasing water level can have negative effect on plant growth due to oxygen shortage (Kotowski et al., 2001; Li et al., 2017), positive effects of shallow groundwater levels on *C. cinerascens* growth suggest that water availability is the main limiting factor for plant growth over seasons and years. Despite not significant, we observed a trend of increasing inflorescence number with higher groundwater level, a finding in agreement with the study of Chen et al. (2015) that reported a positive correlation between the sexual reproduction of *C. brevicuspis* and soil moisture content in Doting Lake wetlands. As Poyang Lake water level is declining due to the effects of Three Gorges Dam, sand mining, and climate change (Lai et al., 2014; Liu, Wu, & Zhao, 2013; Piao et al., 2010; Zhang et al., 2012), our results suggest that deeper groundwater levels would simultaneously reduce vegetative and sexual reproduction with strong impacts on *C. cinerascens* populations in the future. Wetland ecosystem is known as an important carbon sink (Bernal & Mitsch, 2012; Kayranli, Scholz, Mustafa, & Hedmark, 2010) and as *C. cinerascens* population is an important component of Poyang Lake wetland, any alteration in the productivity of *C. cinerascens* may induce cascading effects on wetland functioning.

In line with our third hypothesis, significant interactive effects of groundwater level and season on the different growth parameters of *C. cinerascens* clearly demonstrated that the effect of groundwater level on plant growth was dependent on the season considered and, as expected, the effects occurred mainly in autumn. In spring, temperature was low, resulting in low soil evaporation and plant transpiration, so that precipitation supplied plants with the required water amount for optimal growth (Appendix). This situation could probably explain why deeper groundwater level only marginally reduced the inflorescence number of *C. cinerascens*. Compared to spring, precipitation amount decreased considerably in autumn while the temperature remained high and as a result the groundwater level became the main water source and a limiting factor for plant growth. Our finding is supported by two previous studies which reported that higher groundwater level had no effect on plant biomass in spring, but increased plant biomass in autumn in Dongting Lake wetland, China (Li et al., 2014, 2017). However, after the withering of the plant population in summer, we observed that *C. cinerascens* growth recovered in autumn only under 10 cm groundwater level. From this result, we could deduce that decreasing groundwater level in the studied Poyang Lake wetland will negatively affect *C. cinerascens* regeneration and growth particularly during in autumn.

## 5 CONCLUSION

Our study provides clear evidence that the groundwater level can have distinct effects on plant growth according to the season, with generally stronger effects occurring in autumn compared to spring and summer. Indeed, under low precipitation, as typically observed in autumn, groundwater level controls water availability and becomes an important driver of plant growth, which significantly influences annual biomass production of *C. cinerascens*. Overall, lower groundwater level of Poyang Lake impedes *C. cinerascens* development and constrains population growth but does not affect sexual reproduction in spring, as water availability is ensured by sufficient amount of precipitations. Additionally, our results also showed that, independently of the season and groundwater level, population biomass of *C. cinerascens* was lower during the drier years (i.e., 2016), further highlighting the role of water limitation on plant growth in Poyang Lake. Altogether, these findings suggest that any change in water availability might strongly impact plant communities. Interactive effects of groundwater level and precipitation patterns should therefore be taken into account to predict plant community dynamics in Poyang Lake wetlands.

## CONFLICT OF INTEREST

None declared.

## AUTHOR CONTRIBUTIONS

WF and LX designed and performed the experiment. WF, PM, and MS analyzed the data and led the writing of the manuscript. All authors contributed critically to the drafts and gave final approval for publication.

## Data Availability

Dryad ://doi.org/10.5061/dryad.stqjq2c04.

## Appendix 1

Ombrothermic diagram of the study site during the period from January 2015 to December 2016. Bars represent mean monthly precipitation (mm) and circles represent mean monthly temperature (°C).
